# Do Not Be Fooled by Fancy Mutations: Inflammatory Fibroid Polyps Can Harbor Mutations Similar to Those Found in GIST

**DOI:** 10.1155/2013/845801

**Published:** 2013-11-06

**Authors:** Bodil Bjerkehagen, Kristin Aaberg, Sonja E. Steigen

**Affiliations:** ^1^Department of Pathology, Oslo University Hospital, Norwegian Radium Hospital, Pb 4953 Nydalen, 0424 Oslo, Norway; ^2^Department of Pathology, University Hospital of North Norway, 9038 Tromsø, Norway; ^3^Department of Medical Biology, Faculty of Health Sciences, University of Tromsø, 9037 Tromsø, Norway

## Abstract

*Goal*. Surgeons that remove a typical polyp from the stomach or small intestine should be reluctant to accept a diagnosis of GIST just because there is a mutation in platelet-derived growth factor receptor alfa (*PDGFRA*). *Background*. A subtype of gastric and intestinal polyps is denoted as inflammatory fibroid polyp (IFP). In some of these cases a mutation in *PDGFRA* is found, leading to the diagnosis of gastrointestinal stromal tumor (GIST). *Study*. This study includes two patients that had polyps removed from the ileum, and an extended investigation was performed with immunohistochemical staining and mutation analyses. *Results*. The tumors did not show typical immunohistochemical staining for markers used to diagnose GIST, but the mutation analysis revealed a mutation in *PDGFRA* exon 12. On the basis of the mutation analysis, both polyps were primarily diagnosed as GISTs, but the diagnosis was later changed to inflammatory fibroid polyp. *Conclusion*. It is important that both surgeons and pathologists be aware that IFP can harbor a mutation in *PDGFRA* where further treatment and follow-up is different with the two different diagnoses. A mutation analysis can be misleading when taken out of the context of clinical observations, histological characteristics and immunohistochemical staining.

## 1. Introduction

Gastric and intestinal polyps can broadly be defined as luminal lesions projecting above the plane of the mucosal surface. They are relatively frequent in routine pathology practice, where the main goal is to rule out the possibility of malignancy. Various subtypes of gastric polyps are recognized and generally divided into nonneoplastic and neoplastic with the latter being defined as an abnormal proliferation of cells with some degree of malignant potential.

In 1948 Vanek described gastric polyps that he termed “submucosal granuloma with eosinophilic infiltration” [1]. Subsequently these polyps have had multiple names, but the term inflammatory fibroid polyp (IFP) has gained wide acceptance [2]. They are mostly found in the antrum/pyloric area and in ileum [3, 4] and may be found incidentally or during evaluation of gastric hemorrhage, anemia, or symptoms of gastric outlet obstruction [5, 6]. Inflammatory fibroid polyps are well-circumscribed, solitary, small sessile or pedunculated lesions. The polyps usually do not reoccur after resection and, therefore, local excision is an adequate treatment [7, 8]. 

Histologically the polyps are centered in the submucosa and characterized by the proliferation of small, thin-walled blood vessels surrounded by a mesenchyme of short spindle cells that may be arranged in an “onion-skin” pattern around larger vessels. There are many inflammatory cells, often dominated by eosinophils. Immunohistochemical evaluations show that the tumors are variable and often CD34 positive but CD117 negative [4, 6]. 

Inflammatory fibroid polyps can harbor mutations in the platelet-derived growth factor receptor alfa (*PDGFRA*) gene, and this was first published by Schildhaus et al. [9] and later also by Lasota et al. [4]. Such *PDGFRA *mutations had previously only been found in gastrointestinal stromal tumors (GISTs) [10]. 

GISTs are the most common mesenchymal tumors of the gastrointestinal tract most commonly found in the stomach and small intestine [11]. Morphologically, they are usually cellular tumors composed of spindle or epithelioid cells but can show a wide variety of histological features. Immunohistologically they are usually CD117, DOG1, and CD34 positive with some cases staining for smooth muscle actin, desmin, and S100 [12]. Gain-of-function mutations have been found in the *KIT *gene (type III tyrosine kinase family) or in the *PDGFRA* gene with the two of them being practically mutually exclusive [10, 13]. The GISTs that show mutations in *PDGFRA *are often CD117 negative and with a dominant histological appearance of epithelioid-like cells. It is often considered that molecular analyses are necessary to confirm the diagnosis of GIST and to determine further therapeutic strategy for these patients [14]. 

In this report we consider two cases primarily diagnosed as probable GISTs after histological evaluation, where the molecular analyses were performed to confirm the diagnosis and for therapeutic strategy. A retrospective evaluation of these two cases revealed that the mutational analyses used to confirm the diagnosis of GIST are also applicable for confirming the diagnosis of inflammatory fibroid polyp. 

## 2. Materials and Methods

### 2.1. Cases

Patient number one was a woman in her late thirties that received surgery because of invagination in the ileum and with the discovery of a polyp. She had abdominal pain two days prior to admittance to hospital. The resected small intestine was 18.5 cm long and contained an almost circular polyp with largest diameter 3.8 cm. At the base four small wart-like lesions 0.2–0.5 cm in diameter were found. The lesion was resected with free margins. She had a CT scan 12 months after her operation without any evidence of relapse, and at the clinical consultation she was in good health. 

Patient number two was a man in his late forties with a polyp in the distal ileum. Prior to his operation he had complained of abdominal pain for 7 months. He too was operated because of invagination. The pathologist received 14 cm of small intestine with a freely resected 5 cm long polyp that was 1.4 cm in diameter at the base. The patient did not have any further surgery and did not receive any additional medication. He experienced some nausea a couple of months after the operation, but at control after 5 months he felt healthy. A CT scan at this point did not reveal any pathological features. 

### 2.2. Immunohistochemical Staining

Both cases were evaluated on hematoxylin and eosin stain and in addition were stained immunohistochemically with CD117, DOG1, CD34, ki67, CD45, S100, desmin, and smooth muscle actin. The staining was evaluated independently by two senior pathologists (BB and SES).

### 2.3. Mutational Analyses

For DNA isolation, the lesional areas were marked on a hematoxylin and eosin (H&E) slide by a senior pathologist. The slides were used as a guide to orient the formalin-fixed, paraffin-embedded (FFPE) tissue block, and tumor tissue was selected from the block by a scalpel. Paraffin was removed with xylene, and total DNA was isolated using QIAamp DNA mini kit (Qiagen GmbH, Hilden, Germany). Selected parts of *KIT* exon 9, 11, 13, and 17 and *PDGFRA* exon 12 and 18 were amplified by PCR. Primer sequences have been published previously [13], except for primer sequences for *KIT* exon 13 and 17. These exons were amplified using the following intronic primer pairs (listed 5′ to 3′): exon 13F ATGCGCTTGACATCAGTTTG; exon 13R CATGTTTTGATAACCTGACAGACA; exon 17F GGTTTTCTTTTCTCCTCCAACC; exon 17R TGAAACTAAAAATCCTTTGCAGG. The annealing temperature for all sets of primers was 55°C. The PCR products were analyzed by direct sequencing using a BigDye Terminator (Applied Biosystems, Foster City, CA, USA) and run on an ABI 3130 Genetic Analyzer (Applied Biosystems, Foster City, CA, USA). All PCR products were sequenced on both strands. In addition the PCR products of *KIT* exon 9 and 11 and *PDGFRA* exon 12 and 18 were analyzed by capillary electrophoresis using the ABI 3130 Genetic Analyzer (Applied Biosystems, Foster City, CA, USA) to target insertions or deletions in these exons. The nomenclature of the mutations is based on the recommendations of Human Genome Mutation Society (http://www.hgvs.org/mutnomen/). The *KIT* sequence (NM_000222.1) and the *PDGFRA* sequence (NM_006206) obtained from the National Centre for Biotechnology Information (NCBI) at http://www.ncbi.nlm.nih.gov/ were used as reference sequences. 

## 3. Results

### 3.1. Histology and Immunohistochemistry

Both tumors had an equal histological appearance with many small vessels and a loose stroma with many inflammatory cells. Many of these were eosinophilic granulocytes. The stroma was negative for the GIST markers CD117 and DOG1 and the neuroderived marker S100. The mesenchymal marker CD34 was negative, but there was a weak stain with smooth muscle actin. Desmin was only positive in the vessel walls. CD45 was positive in inflammatory cells. The proliferation marker Ki67 was positive in around 10% of the tumor cells. 

### 3.2. Mutation Analysis

**Figure 1:**
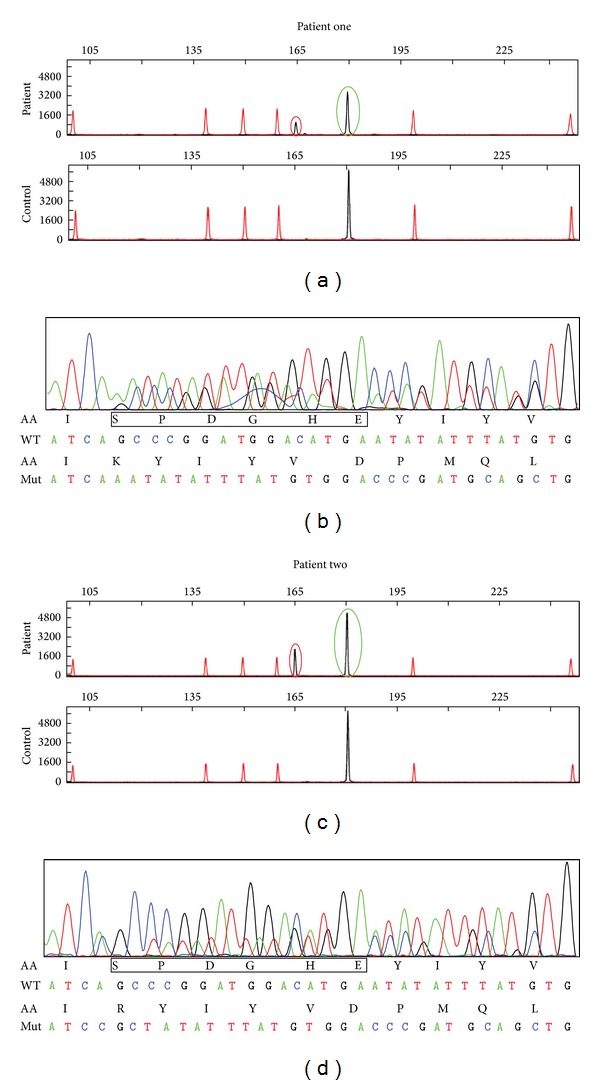
Capillary electrophoresis ((a) and (c)) and direct sequencing ((b) and (d)) of *PDGFRA* exon 12 from patient numbers one and two. Red rings indicate mutated alleles; green rings indicate normal alleles. Black boxes indicate amino acids deleted in the mutated alleles.

 Patient one: c.1696_1711del (p.Ser566_Glu571delinsLys)
 Patient two: c.1696_1713delinsCGC (p.Ser566_Glu571delinsArg) Figure 1.

## 4. Discussion

IFP is a benign neoplasm that should not be mistaken for a GIST. The treatment of IFP is surgery only, and fully resected polyps hardly reoccur. Gastrointestinal stromal tumors differ from the IFP by having a malignancy potential even at small size and lesions arising in the small bowel are graded as more aggressive than those found in the stomach [15]. 

Clinically the IFP is an intraluminal polyp in the stomach or small intestine and is often simply regarded by the surgeon as a polyp. The GIST arises from the cells of Cajal in the intramuscular part of the GI-wall and is mostly found as a tumor under the mucosa. The histological picture of the IFP reveals a tumor with plenty of small vessels surrounded by loose mesenchyme containing a prominent inflammatory infiltrate. Many of the inflammatory cells are eosinophilic granulocytes. The GIST is in most cases composed of spindle cells arranged in fascicles or sheets but can also reveal more epithelioid features. They can be highly vascularized, but mostly they have small and inconspicuous vessels. Some cases have quite a few inflammatory cells, but mostly they contain only scattered lymphocytes. Immunohistochemically, IFPs are variably positive for CD34 and negative for CD117 and DOG1, while GISTs are almost always positive for CD117, DOG1, and CD34. Both tumors are mostly negative for actin, desmin, and S100. Based on this, the diagnosis seems to be quite straight forward, but sometimes the question of GIST is raised even if the clinical information, histology, or the immunohistochemical staining does not indicate this. This is based on the fact that GIST can display many different clinical and histological patterns, and a mutation in *KIT* or *PDGFRA *is believed to confirm the diagnosis. 

Approximately 10% of GISTs are wild type, and without a mutation the diagnosis of GIST relies on expression of immunohistochemical markers. In around 90% of the GISTs a mutation in *KIT* or *PDGFRA* is found. A mutation in *PGDFRA* exon 12 is very rarely found in GISTs, with an incidence of around 2% [13]. Lasota et al. found, however, mutations in *PDGFRA *12 and 18 in 55% of 60 investigated inflammatory fibroid polyps, with more than 90% of these being in *PDGFRA* 12 [4]. 

The presented two cases in ileum did not have typical histopathological features or immunohistochemical staining expression patterns typical for GIST, but the clinicians had raised the question about GIST. To reassure them and ourselves that the patients had received the right diagnosis, mutation analyses were performed. On the base of the mutation analyses of the first case we concluded, reassuringly, that the diagnosis of GIST was correct. When case two came, sometime later, and also had this rare mutation in *PDGFRA* exon 12, the diagnosis was not so obvious anymore. A literature search revealed that IFP can harbor just such mutations, and both diagnoses were later revised from GIST to IFP.

The clinicians removed a polyp and did not question the diagnosis of GIST after the pathologist had supplemented the analysis with a convincing mutation analysis. The pathologists were puzzled by the histopathological appearance, but the mutation analyses were unmistakable. We were all fooled to believe that the two cases were GISTs on the basis of the *PDGFRA* exon 12 mutation. Technological progress can be of invaluable help but can also create confusion and lead to wrong conclusions when results are misinterpreted. A combination of clinical information, histopathological features, immunohistochemical staining, and molecular analyses is essential for making the correct diagnosis. 

## References

[1] Vanek J (1949). Gastric submucosal granuloma with eosinophilic infiltration. *American Journal of Pathology*.

[2] Helwig EB, Ranier A (1953). Inflammatory fibroid polyps of the stomach. *Surgery, Gynecology & Obstetrics*.

[3] Wysocki AP, Taylor G, Windsor JA (2007). Inflammatory fibroid polyps of the duodenum: a review of the literature. *Digestive Surgery*.

[4] Lasota J, Wang Z-F, Sobin LH, Miettinen M (2009). Gain-of-function PDGFRA mutations, earlier reported in gastrointestinal stromal tumors, are common in small intestinal inflammatory fibroid polyps. A study of 60 cases. *Modern Pathology*.

[5] Kolodziejczyk P, Yao T, Tsuneyoshi M (1993). Inflammatory fibroid polyp of the stomach: a special reference to an immunohistochemical profile of 42 cases. *American Journal of Surgical Pathology*.

[6] Hasegawa T, Yang P, Kagawa N, Hirose T, Sano T (1997). CD34 expression by inflammatory fibroid polyps of the stomach. *Modern Pathology*.

[7] Lewin KJD, Appelman H (1996). Mesenchymal tumors and tumor-like proliferation. *Tumors of the Esophagus and Stomach*.

[8] Paikos D, Moschos J, Tzilves D (2007). Inflammatory fibroid polyp or Vanek’s tumour. *Digestive Surgery*.

[9] Schildhaus H-U, Caviar T, Binot E, Büttner R, Wardelmann E, Merkelbach-Bruse S (2008). Inflammatory fibroid polyps harbour mutations in the platelet-derived growth factor receptor alpha (PDGFRA) gene. *Journal of Pathology*.

[10] Heinrich MC, Corless CL, Duensing A (2003). PDGFRA activating mutations in gastrointestinal stromal tumors. *Science*.

[11] Miettinen M, Lasota J (2003). Gastrointestinal stromal tumors (GISTs): definition, occurrence, pathology, differential diagnosis and molecular genetics. *Polish Journal of Pathology*.

[12] Steigen SE, Eide TJ (2006). Trends in incidence and survival of mesenchymal neoplasm of the digestive tract within a defined population of Northern Norway. *APMIS*.

[13] Steigen SE, Eide TJ, Wasag B, Lasota J, Miettinen M (2007). Mutations in gastrointestinal stromal tumors—a population-based study from northern Norway. *APMIS*.

[14] Yamamoto H, Kojima A, Nagata S, Tomita Y, Takahashi S, Oda Y (2011). KIT-negative gastrointestinal stromal tumor of the abdominal soft tissue: a clinicopathologic and genetic study of 10 cases. *American Journal of Surgical Pathology*.

[15] Joensuu H, Vehtari A, Riihimäki J (2012). Risk of recurrence of gastrointestinal stromal tumour after surgery: an analysis of pooled population-based cohorts. *The Lancet Oncology*.

